# PPARα and PPARβ/δ are negatively correlated with proinflammatory markers in leukocytes of an obese pediatric population

**DOI:** 10.1186/s12950-020-00264-2

**Published:** 2020-10-31

**Authors:** Karina Vargas-Sánchez, Laura Vargas, Yenny Urrutia, Iván Beltrán, Ana Beatriz Rossi, Hernán Yupanqui Lozano, Jorge Guarín, Monica Losada-Barragán

**Affiliations:** 1 Research group of Translational Neurosciences, School of Medicine, Universidad de los Andes, Bogotá, 111711 Colombia; 2 Biología celular y funcional e ingeniería de biomoléculas, Universidad Antonio Nariño, Bogotá, Colombia; 3 GRINCIBIO. Universidad Antonio Nariño, Bogotá, Colombia; 4 DEXA DIAB, Bogotá, Colombia

**Keywords:** Obesity, Children, Adolescents, PPAR, GLP-1R, Cytokines

## Abstract

**Background:**

Obesity configures a pathophysiological profile that predisposes the development of metabolic and cardiovascular diseases, critically impacting public health. The chronic dysregulation of immuno-metabolic components triggered by pediatric obesity is a common but scarcely understood aspect of the disease. Peroxisome proliferator-activated receptors (PPARs) are a group of transcription factors essential for energy and immune homeostasis of different tissues. Besides, the glucagon-like peptide-1 receptor (GLP-1R) activation influences insulin secretion, but also regulates the cytokine profile possibly mediated through a PPAR isotype. However, the role of PPARs and GLP-1R in leukocytes from obese pediatric patients remains unclear. Therefore, we examined the expression of PPARs isotypes and GLP-1R in leukocytes, and its correlation with metabolic, hormonal, inflammatory, and anthropometric markers in an obese pediatric population.

**Results:**

Obese children and adolescents presented a significant increase in anthropometric and body composition parameters, TG, VLDL, TG/HDL, android fat (%)/gynoid fat (%) (A/G%) index, and HOMA score when compared with the control group. Obese participants exhibited a pro-inflammatory profile with an augment of IL-8 (*p* = 0,0081), IL-6 (*p* = 0,0005), TNF-α (*p* = 0,0004), IFN-γ (*p* = 0,0110), MCP-1 (*p* = 0,0452), and adipsin (*p* = 0,0397), whereas displayed a reduction of adiponectin (*p* = 0,0452). The expression of PPARα and GLP-1R was lower in the leukocytes from obese participants than in lean subjects. Furthermore, PPARα correlates negatively with TNF-α (*p* = 0,0383), while GLP-1R did not show correlation with any inflammatory variable. However, both receptors correlate negatively with the abdominal skinfold. Although PPARβ/δ expression was similar between groups, it was negatively associated with IL-8 levels (*p* = 0,0085).

**Conclusions:**

PPARα and PPARβ/δ expression are negatively correlated with the proinflammatory markers TNF-α and IL-8, respectively, suggesting participation in the regulation of inflammation which was observed to be altered in pediatric obesity. Furthermore, PPARα and GLP-1R are downregulated in leukocytes from obese participants. The low expression of both receptors is correlated with an increase in abdominal skinfold, suggesting a role in fat distribution that could indirectly affect cytokine secretion from different immune and adipose cells, likely triggering an inflammatory profile as a consequence of obesity. Altogether, these findings may impact the understanding and implementation of PPARα or GLP-1R agonists in the clinic.

**Supplementary Information:**

The online version contains supplementary material available at 10.1186/s12950-020-00264-2.

## Background

Obesity is a chronic disease with a high impact on public health due to its increased prevalence. As a result of obesity, adult subjects can develop type 2 diabetes (T2D) and cardiometabolic diseases. Nowadays those events are not limited to adults, but rather they are remarkably increased in children and adolescents with obesity [1]. T2D at early life stages carries on into adulthood, which is related to a more severe damage of β-cells [2], and to increased appearance of additional comorbidities such as cardiovascular disease, kidney and visual failure [3, 4].

The metabolic phenotype in obesity is determined by alterations in metabolic regulation of triglycerides and glucose, which can lead to a chronic inflammatory status characterized by the enriched circulation of proinflammatory cytokines [5–8]. The inflammatory profile displayed in obese children and adolescents is frequently associated with insulin resistance (IR), hyperinsulinemia, hyperglycemia, and metabolic syndrome, gradually leading to the development of T2D and cardiometabolic diseases [9]. Noticeably, the study of metabolic sensors that regulates the immune proinflammatory response in obesity remains poorly assessed.

Peroxisome proliferator-activated receptors (PPARs) are recognized transcription factors, comprising a group of three isotypes: PPARα, PPARβ/δ, and PPARγ. They play an essential role in regulating the expression of genes responsible for lipid and carbohydrate metabolism, as well as maintenance mitochondrial metabolic rate [10]. PPARs participate in the regulation of the expression of the cytokine profile from adipose tissue and immune cells with a proven anti-inflammatory role [8, 11].

An additional factor that can display a dual role in metabolism and immunity is the glucagon-like peptide-1 (GLP-1)/GLP-1R axis. GLP-1 is a hormonal factor that canonically induced an increase in the pancreatic release of insulin [12, 13]. This axis showed a cytoprotective role in pancreatic β-cells, delaying the establishment of obese T2D [12, 14]. A non-canonical effect of GLP1 is associated with the activation of GLP-1R to modulate cytokine production. GLP-1R signaling on invariant natural killer T cells (iNKT) can enhance the expression of anti-inflammatory cytokines such as IL-10 [12]. Besides, GLP-1R also downregulates pro-inflammatory cytokines expression, mediated through PPARγ activation and followed by NF-kB inhibition [15].

The regulatory genes characterization of metabolic and inflammatory processes can be accomplished in leukocytes. Leukocytes continuous metabolic sensing significantly contributes to the coordination of organism-wide metabolic and immunological responses [16]. Venous blood obtention from humans is a minimally invasive procedure and represents a widely accessible source which can be easily assessed to identify gene expression in leukocytes. Thus, blood analyses are valuable for early diagnosis and opportune intervention for both immune and metabolic disorders. Remarkably, few studies in obese population focus on PPARs isotypes and GLP-1R in leukocytes. Therefore, the aim of this study is to evaluate the expression of PPARs and GLP-1R in leukocytes, and its association with inflammatory, metabolic, body composition, and anthropometric factors in an obese pediatric population.

## Results

### Obese children and adolescents have an impaired metabolic and anthropometric profile

The anthropometric, body composition and biochemical variables of the studied individuals stratified by body mass index (BMI) percentile status are shown in Table 1. Age and gender of the participants were not significantly different among the control and obese group (Additional files 3 and 4). The median age was 11 years old, and 48% were girls. All mean values of skinfold thickness*, waist circumference* (WC), *arm circumference* (AC), *waist-to-height ratio* (WHR), *arm muscle circumference,* and *neck circumference* were significantly increased in participants with obesity in comparison with healthy weight children and adolescents (Table 1). Regarding body composition, the *percentage of arm, leg, trunk, android, gynoid,* and *body fat* were significantly elevated in obese patients compared with control subjects (Table 1). In obese children, fat distribution was similar between different body areas, and there were no significant differences in mean values of *lean mass* between groups.

**Table 1:** Anthropometric and body composition parameters in children and adolescents^a^

	Control	Obesity	***p*** value
	*n* = 19	*n* = 18	
**Gender**			
**Female**	8	10	
**Male**	11	8	
**Age (years)**	10,47 ± 2,87	11,33 ± 2,93	0,3515
**BMI percentile**	49,92 ± 28,03	96,37 ± 8,50	< 0,0001**
**Arm circumference (cm)**	20,41 ± 3,23	27,45 ± 3,50	< 0,0001**
**Arm muscle circumference (cm)**	16,77 ± 2,83	20,64 ± 3,19	0,0008*
**Neck circumference (cm)**	28,29 ± 3,03	32,47 ± 2,82	0,0003*
**Triceps skinfold (cm)**	11,68 ± 4,61	21,64 ± 4,94	< 0,0001**
**Subscapular skinfold (cm)**	6,85 ± 2,27	21,17 ± 5,40	< 0,0001**
**Suprailiac skinfold (cm)**	10,91 ± 4,78	26,61 ± 5,79	< 0,0001**
**Abdominal skinfold (cm)**	10,53 ± 5,01	27,64 ± 6,17	< 0,0001**
**Waist circumference (WC) (cm)**	63,75 ± 7,53	83,75 ± 6,94	< 0,0001**
**Waist/Height ratio (WHR)**	0,45 ± 0,03	0,58 ± 0,04	< 0,0001**
**Arm fat (%)**	33,07 ± 9,44	45,91 ± 7,73	< 0,0001**
**Leg fat (%)**	32,82 ± 7,74	44,41 ± 5,82	< 0,0001**
**Trunk fat (%)**	21,06 ± 8,95	42,9 ± 6,79	< 0,0001**
**Android fat (%)**	18,58 ± 9,93	44,33 ± 7,53	< 0,0001**
**Gynoid fat (%)**	28,88 ± 8,21	44,68 ± 5,96	< 0,0001**
**Body fat (%)**	26,28 ± 7,35	39,9 ± 5,89	< 0,0001**
**Lean mass (kg)**	32,83 ± 10,31	22,84 ± 11,25	0,0073*

Values are presented as percentage or mean ± standard deviation (SD). Differences between groups were evaluated using Wilcoxon signed-rank tests

*p <  0.005, ** *p* <  0.0001

^a^*BMI* body mass index

The mean of fasting glucose and 2-h glucose fell within the normal ranges, as recommended by the American Academy of Pediatrics (Table 2**)** [17]**.** However, insulin was significantly higher in children with obesity than in the standard weight group. Accordingly, the HOMA score was increased in obese children; this score overcomes the 3,4 cut-off indicating IR (Table 2) [18]. TG and VLDL levels were higher in obese children than those of lean subjects, whereas HDL-c levels were significantly reduced in obese subjects in contrast with the control group (Table 2). The levels of total cholesterol were similar among groups (Table 2).

**Table 2:** Biochemical and hormonal variables in children and adolescents^a^

	Control	Obesity	***p*** value
	*n* = 19	*n* = 18	
**Fasting blood glucose (mg/dL)**	87,61 ± 5,55	87,59 ± 6,25	0,936
**2 h glucose (mg/dL)**	91,74 ± 13	94,23 ± 13,58	0,7617
**Insulin (mcU/mL)**	7,77 ± 4,78	19,77 ± 8,02	< 0,0001**
**Total cholesterol (mg/dL)**	150,15 ± 21,83	158,29 ± 24,91	0,3758
**Triglycerides (mg/dL)**	76,48 ± 18,47	117,27 ± 31,64	0,0001*
**HDL (mg/dL)**	50,34 ± 8,22	41,39 ± 9,51	0,0077*
**LDL (mg/dL)**	84,55 ± 18,04	94,70 ± 20,67	0,2547
**VLDL (mg/dL)**	15,29 ± 3,69	23,45 ± 6.33	0,0001**
**HOMA-IR (score)**	1,50 ± 1,19	4,28 ± 1,72	< 0,0001**
**TG/HDL ratio**	1,60 ± 0,61	3,02 ± 1,24	< 0,0001**
**Android-gynoid % fat ratio**	0,61 ± 0,17	0,99 ± 0,08	< 0,0001**
**GLP-1 (ng/mL)**	15,98 ± 0,69	17,70 ± 0,66	0,0725
**GIP (pg/mL)**	2637 ± 486,89	3137 ± 512,44	0,6299

Values are presented as percentage or mean ± standard deviation (SD). Differences between groups were evaluated using Wilcoxon signed-rank tests

* *p* < 0.005, ** *p* < 0.0001

^a^*HDL* high-density lipoprotein, *VLDL* very low-density lipoprotein, *LDL* low-density lipoprotein, *HOMA-IR* homeostatic model of assessment for insulin resistance, *GLP-1* glucagon-like peptide 1, *GIP* gastric inhibitory polypeptide

*TG/HDL* and *A/G ratios* are frequently used as indicators of cardiometabolic risk, and here, those ratios were significantly elevated in the obese group in comparison with the lean patients. Based on this data, obese children and adolescents have an increased risk of developing cardiovascular disease and T2D. Incretins gastric inhibitory polypeptide (GIP) and GLP-1 did not show significant differences between the study groups (Table 2).

### Triceps skinfold displayed a positive correlation with body composition parameters

Relationships between fat body distribution and anthropometric data are shown in Table 2. *Triceps skinfold* displayed a positive correlation with all body composition parameters; the stronger correlation was observed with *total body fat percentage* (*r* = 0,6554, *p* = 0,0071), while a weaker association with *Android fat percentage* (*r* = 0,4974, *p* = 0,0516) was found (Table 3). Furthermore, *WHR ratio* showed a positive association with *arm* (*r* = 0,691, *p* = 0,0038)*, leg* (*r* = 0,5245, *p* = 0,0388)*, android* (*r* = 0,5528, *p* = 0,0282), *and total body fat percentages* (*r* = 0,5379, *p* = 0,0334) (Table 3).

**Table 3:** Correlation of DEXA fat measurements with anthropometric data^a^

	Arm fat (%)	Leg fat (%)	Trunk fat (%)	Android fat (%)	Gynoid fat (%)	Body fat (%)
**BMI**	0,060	0,131	− 0,013	− 0,063	− 0,028	0,081
**Arm circumference**	− 0,118	0,014	− 0,167	− 0,217	− 0,179	− 0,046
**Arm muscle circumference**	− 0,340	− 0,202	− 0,437	− 0,419	− 0,425	− 0,299
**Neck circumference**	− 0,449	− 0,278	− 0,411	− 0,496	− 0,391	− 0,355
**Triceps skinfold**	0,590*	0,627*	0,604*	0,497*	0,579*	0,655*
**Subscapular skinfold**	0,148	0,022	0,294	0,186	0,179	0,171
**Suprailiac skinfold**	− 0,041	− 0,186	0,099	0,140	0,031	−0,027
**Abdominal skinfold**	0,189	−0,029	0,117	0,289	−0,004	0,119
**Waist circumference (WC)**	−0,105	−0,110	− 0,255	−0,217	− 0,234	−0,149
**Waist/Height ratio (WHR)**	0,691**	0,525*	0,442	0,553*	0,482	0,538*

* *p* < 0.05 and ** *p* < 0.005

^a^ BMI: body mass index. Spearman’s rank correlation test

*Trunk fat percentage* presented a significant association with *A/G ratio* (*r* = 0,558, *p* = 0,027). Likewise, *Android fat percentage* also displayed a positive correlation with *A/G ratio* (*r* = 0,558, *p* = 0.006). *Arm circumference* parameter correlated negatively with LDL (*r* = − 0,572, *p* = 0,013) and no-HDL (*r* = − 0,502, *p* = 0,034), whereas *arm muscle circumference* exhibited a weak negative correlation with LDL (*r* = − 0,486, *p* = 0,041) (Additional file 2).

The correlation of different variables with the *suprailiac skinfold* indicated a positive association with *Total Cholesterol* (*r* = 0,658, *p* = 0,003), and *no-HDL* (*r* = 0,724, *p* = 0,001), and a weaker association with *LDL* (*r* = 0,550, *p* = 0,018) (Additional file 2). Also, there was a positive correlation between *Abdominal skinfold* and *HOMA-IR* (*r* = 0,459, *p* = 0,055).

### *PPARα* and *GLP-1R* gene expression is reduced in leukocytes from obese subjects

**Fig. 1:**
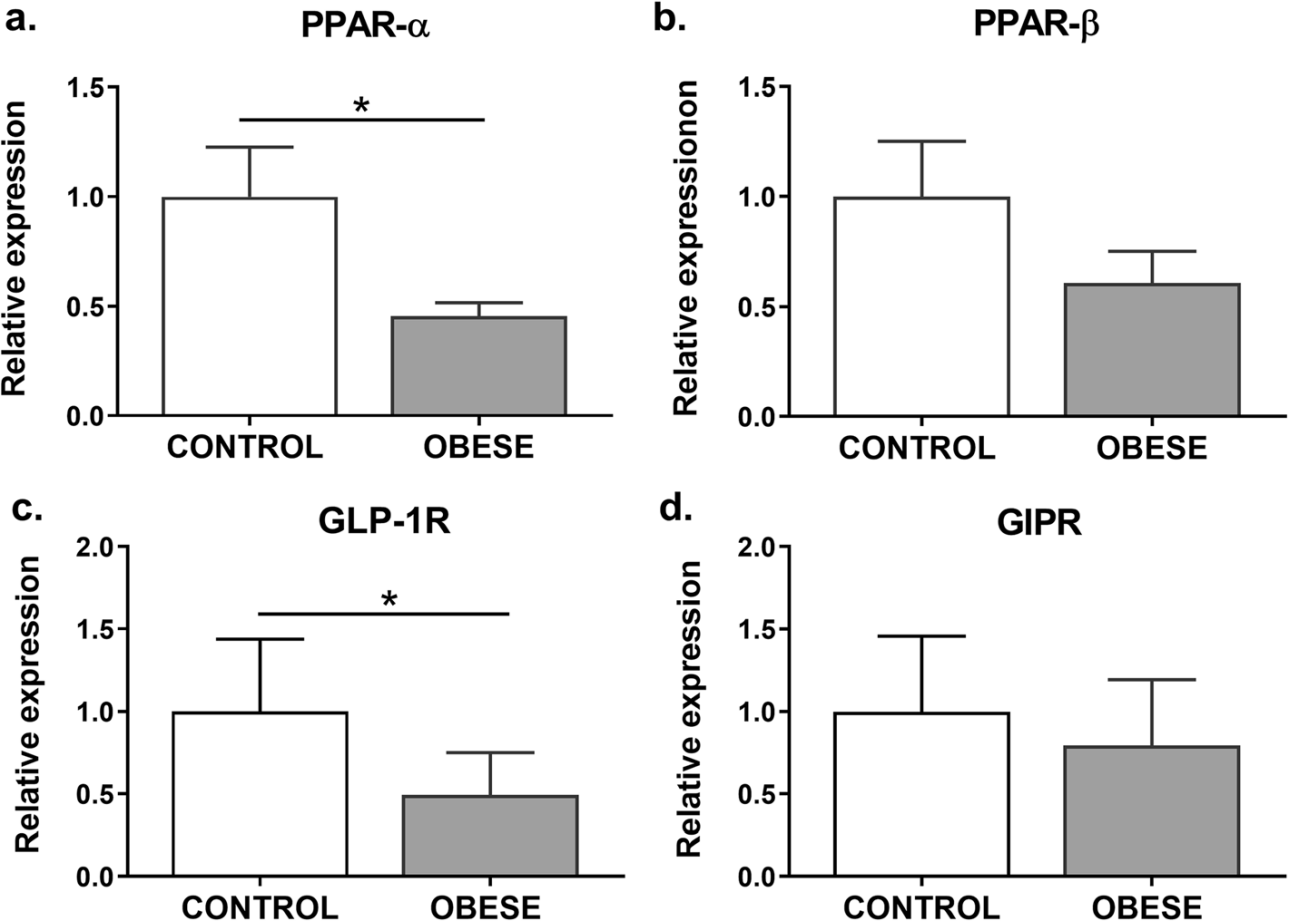
Leukocytes from obese children and adolescents displayed a reduction of *PPAR-α* and *GLP-1R* gene expression. **a** *PPAR-α*, **b** *PPAR-β*, **c** *GLP1R,* and **d** *GIPR* mRNA expression levels were measured by qPCR in leukocytes from each group. The values are expressed as a normalized ratio between the target gene expression and the geometric median of the *PGK* and *YWHAZ* endogenous genes (*n* = 19 for obese group, *n* = 18 for lean group). Statistical differences were analyzed by Student’s *t* test (* *p* <  0.05)

To determine the expression of PPAR isotypes in the leukocytes, the expression of each isotype was analyzed by qPCR. *PPAR-α* expression showed a significant reduction (about 50%) in the leukocytes from obese patients in comparison with the control group (*p* = 0,0484) (Fig. 1a). *PPAR-β* showed no differential expression between the study groups (Fig. 1b). In contrast, *PPAR-γ* expression was undetectable in leukocytes from all the samples. To corroborate this finding, we used adipose tissue cDNA as a positive control, since this isotype is mainly present in adipose cells. This sample presented a positive amplification and a single peak in the dissociation curve, indicating that the reaction was specific (data not shown). Likely, *PPAR-γ* transcripts were not expressed on leukocytes or were expressed at low levels but just in some cell lineages of these samples.

Besides, analysis from mRNA expression levels of incretin receptors was also evaluated in the studied individuals. *GLP-1R* expression was significantly reduced in the obese group compared with the healthy weight participants (*p* = 0,1358) (Fig. 1c), whereas no difference was observed in *GIPR* expression between the two groups (Fig. 1d).

### Obese children and adolescents showed a proinflammatory profile of adipokines and cytokines

**Fig. 2:**
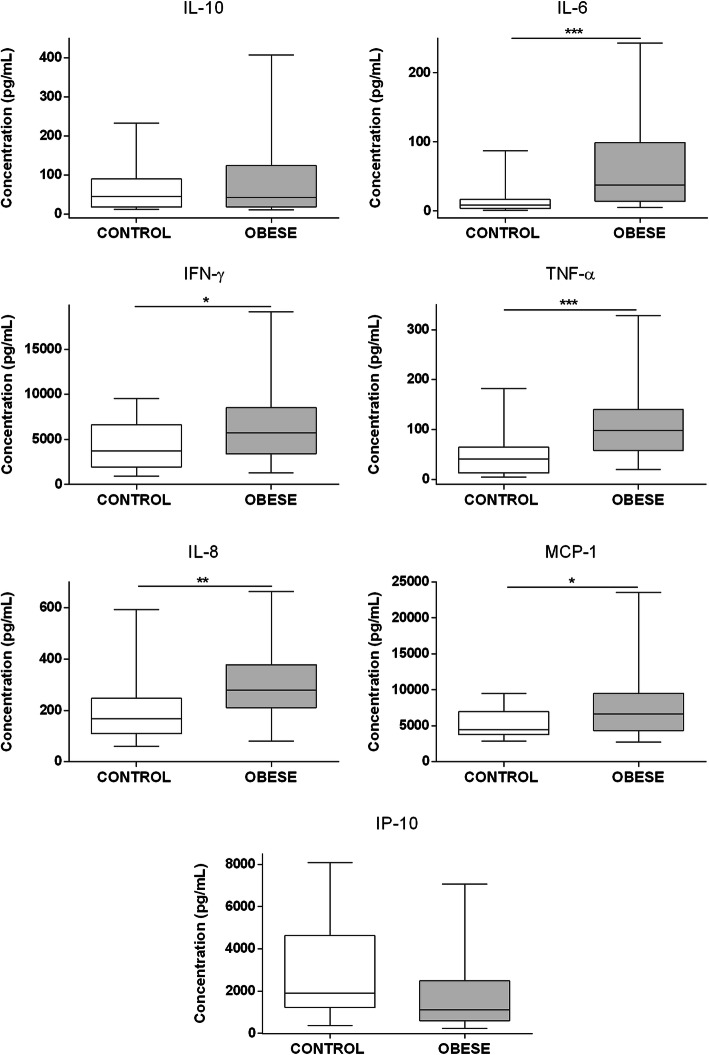
Serum proinflammatory cytokines and chemokines are increased in children and adolescents with obesity. IL-8, IL-6, TNF-α, IFN-γ, IL-10, MCP-1 and IP-10 serum levels were measured by flow cytometry using a Legendplex multiplex assay (*n* = 19 for obese group, *n* = 18 for lean group). Statistical differences were analyzed by Mann Whitney test (* *p* <  0.05, ** *p* <  0.005, *** *p* <  0.0005)

To identify the inflammatory serum profile in obese children and adolescents, the levels of several adipokines and cytokines were measured by flow cytometry using a multiplex assay. Obese subjects showed a significant increase in the levels of IL-8 (*p* = 0,0081), IL-6 (*p* = 0,0005), TNF-α (*p* = 0,0004), IFN-γ (*p* = 0,0110), and MCP-1 levels (*p* = 0,0452), compared with the normal weight group (Fig. 2). Serum levels of IL-10 and IP-10 were detected but did not differ significantly between the groups.

**Fig. 3:**
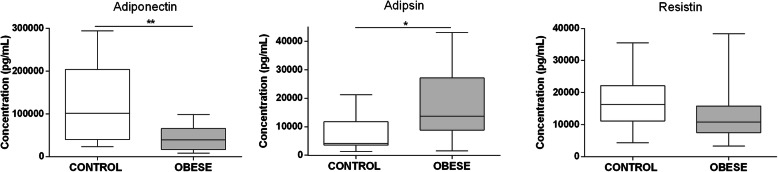
Serum proinflammatory adipokines are increased in children and adolescents with obesity. Adiponectin, adipsin and resistin serum levels were measured by flow cytometry using a Legendplex multiplex assay (*n* = 19 for obese group, *n* = 18 for lean group). Statistical differences were analyzed by Mann Whitney test (* *p* <  0.05, ** *p* <  0.005, *** *p* <  0.0005)

The serum adiponectin concentration presented a significant reduction in the obese group (*p* = 0,0452) compared with the control group (Fig. 3). Conversely, adipsin displayed a significantly higher concentration (*p* = 0,0397) in the obese group in comparison with the control group, which had a concentration of 7688 ± 6116 pg/mL (Fig. 3). Resistin showed similar levels between the study groups, with concentrations of 13,102 ± 8342 pg/mL and 16,792 ± 8302 pg/mL for the obese and control participants, respectively (Fig. 3). Finally, Retinol-binding protein 4 (RBP4) did not present a representative concentration in any of the study groups, indicating that it is not secreted in serum at detectable levels under the evaluated conditions.

### PPARα and PPARβ/δ correlate negatively with proinflammatory markers

Analysis of the relationships between *PPAR-α, PPAR-β,* and *GLP-1R* expression, and the levels of cytokines, chemokines, and anthropometric parameters of the obese subjects, showed that *PPAR-α* transcript levels had a significant negative correlation with TNF-α levels (*r* = − 0,583, *p* = 0,03883) (Table 4), as well as with *abdominal skinfold* (*r* = − 0,712, *p* = 0,0016). Besides, *PPAR-β* showed a significant negative correlation with IL-8 levels (*r* = − 0,667, *p* = 0,0085) and with *arm fat percentage* (*r* = − 0,651, *p* = 0,0132) (Tables 4 and 5).

**Table 4:** Correlation between cytokines with *PPAR-α, PPAR-β and GLP-1R* expression in children and adolescents^a^

	***PPAR-α***	***PPAR-β***	***GLP-1R***
**MCP-1**	0,324	0,011	0,235
**IP-10**	0,370	−0,300	0,033
**IL-10**	−0,125	0,000	−0,136
**IL-8**	0,233	−0,664**	−0,007
**IL-6**	−0,343	−0,200	0,317
**IFN-γ**	0,130	−0,411	−0,130
**TNF-α**	−0,583*	−0,059	0,304

* p = 0.0383, ** *p* = 0.0085

^a^ Spearman’s rank correlation test

**Table 5:** Correlation between anthropometric and biochemical parameters with gene expression of metabolic markers in children and adolescents^a^

	***PPAR-α***	***p value***	***PPAR-β***	***p value***	***GLP-1R***	***p value***
**BMI**	0,049	0,854	−0,257	0,354	−0,086	0,773
**Arm fat (%)**	0,052	0,850	−0,651**	0,013	−0,364	0,246
**Leg fat (%)**	0,208	0,438	−0,515	0,061	−0,266	0,404
**Trunk fat (%)**	0,096	0,723	−0,436	0,118	−0,322	0,309
**Android fat (%)**	−0,028	0,915	−0,339	0,231	−0,420	0,177
**Gynoid fat (%)**	0,108	0,690	−0,513	0,062	−0,189	0,546
**Body fat (%)**	0,096	0,723	−0,511	0,063	−0,343	0,276
**Arm circumference**	−0,094	0,713	0,306	0,266	−0,147	0,608
**Arm muscle circumference**	−0,173	0,501	0,411	0,128	0,042	0,892
**Waist circumference (WC)**	−0,308	0,226	0,050	0,860	0,059	0,844
**Neck circumference**	−0,048	0,852	0,190	0,496	0,090	0,758
**Waist/Height ratio (WHR)**	−0,262	0,291	−0,448	0,090	−0,326	0,241
**Triceps skinfold**	0,210	0,415	−0,390	0,146	−0,277	0,330
**Subscapular skinfold**	0,142	0,586	−0,225	0,413	0,183	0,527
**Suprailiac skinfold**	−0,429	0,084	0,285	0,301	−0,236	0,405
**Abdominal skinfold**	−0,712*	0,002	0,379	0,163	−0,678***	0,009
**Fasting blood glucose**	−0,364	0,149	0,340	0,214	−0,279	0,333
**2 h glucose**	0,042	0,876	− 0,412	0,125	− 0,051	0,868
**Insulin**	−0,185	0,471	0,293	0,287	−0,288	0,318
**Total cholesterol**	−0,021	0,934	0,075	0,790	0,209	0,473
**Triglycerides**	−0,247	0,335	−0,064	0,815	−0,125	0,671
**HDL**	0,416	0,098	−0,054	0,845	0,354	0,215
**VLDL**	−0,248	0,333	−0,064	0,815	−0,117	0,685
**LDL**	0,033	0,900	−0,086	0,756	0,077	0,797
**HOMA-IR**	−0,367	0,146	0,343	0,209	-0,341	0,234
**TG/HDL ratio**	-0,396	0,115	0,097	0,731	-0,182	0,532

Spearman’s rank correlation test

* *p* = 0.0016, ** *p* = 0.0132, *** *p* = 0.009

^a^ *BMI* body mass index, *HDL* high-density lipoprotein, *VLDL* very low-density lipoprotein, *LDL* low-density lipoprotein, *HOMA-IR* homeostatic model of assessment for insulin resistance

In contrast, *GLP-1R* expression did not correlate with any inflammatory parameters (Table 4). However, *GLP-1R* showed a negative correlation with *Abdominal skinfold* (*r* = − 0,678, *p* = 0,009) (Table 5).

## Discussion

Childhood and adolescence are critical periods where fundamental metabolic and hormonal changes start and have an impact on adulthood health. Excess weight at these life stages is a critical factor for the onset of cardiometabolic disease and T2D. Thus, those early stages are vital periods for timely intervention. We evaluated the expression of essential metabolic and immune sensor genes, such as isotypes of *PPAR* and *GLP-1R* in leukocytes, and their correlation with metabolic, immune, and anthropometric factors in a Colombian’s pediatric population with obesity.

PPARs participate in the control of the inflammatory process produced by obesity, modulating the expression of proinflammatory cytokines in adipose cells [10]. However, other tissues can contribute to the inflammatory process. Studies of PPARs, particularly of PPARβ/δ and PPARα, on leukocytes from obese children and adolescents, are scarce. However, the assessment of PPARs expression and relation with variables associated with obesity represents an accessible tool for a better comprehension of obesity pathophysiology, and for the development of improved therapeutic strategies [19].

In this work, we identified the expression of PPARα and PPARβ/δ, but not PPARγ in leukocytes from obese children and adolescents. The activation of PPARβ/δ has been mainly studied in “endurance-exercise mimetics” to reduce inflammation as an obesity treatment, particularly focused on the liver, skeletal muscle, and adipose tissue [20, 21]. Moreover, PPARβ/δ is involved in lipid metabolism, regulation of fatty acid oxidation (FAO), inflammation, and modulation of glucose and cholesterol levels [22]. Direct activation of this isotype improves insulin sensitivity and disorders associated with metabolic syndrome in humans [23]. In this study, PPARβ/δ did not show significant differences between the standard weight and obese groups. However, this gene displayed a significant negative correlation with IL-8 levels and *arm fat percentage*. IL-8 (CXCL8) is a proinflammatory chemokine which mediates the crosstalk between obesity and cardiovascular disease [24, 25]. IL-8 is mainly secreted by adipose tissue, fibroblasts, endothelial cells, monocytes, and macrophages when exposed to IL-1β, TNF-α, or LPS [26]. Indeed, we detected a significant increase in TNF-α levels from the serum of obese subjects, which could contribute to the IL-8 secretion in those individuals.

Although the direct link of PPARβ/δ to IL-8 is suggestive, some studies have already related the activation of PPARβ/δ with the modulation of expression of IL-8 in endothelial cells and in endometrium participating in inflammation [27–29]. In line with this evidence, our results suggest that a decrease in the PPARβ/δ expression in leukocytes might consequently augment IL-8 levels and, thereby, the inflammatory response during obesity. Indeed, experiments in mice have associated the deletion in the expression of PPARβ/δ, with a reduction in the number of hepatic M2 macrophages, which results in alterations of lipid metabolism, IR, and decreased expression of anti-inflammatory cytokines [30]. Those findings indicate a protective role of PPARβ/δ in the control of lipid metabolism and the development of inflammatory processes.

On the other hand, PPARα acts as a lipid sensor actively expressed in tissues with high metabolic rates, such as the liver, heart, muscle, kidney, intestinal mucosa, and brown adipose tissues [31, 32]. The use of natural ligands or agonists for this isotype has revealed its participation in glucose homeostasis, IR development, inflammation, atherogenesis, and in the expression of genes related to lipid metabolism [33, 34]. In leukocytes, PPARα expression has barely been studied. However, the expression of PPARα has been reported in immune cells such as monocytes/macrophages, peripheral blood mononuclear cells (PBMCs), and CD4^+^ T cells [10, 35, 36]. Also, subjects with metabolic syndrome significantly decreased PPARα expression in eosinophils by approximately 21% [37]. Our pediatric obese population showed reduced expression of this isotype in leukocytes when compared to the lean group. Likewise, studies on mice with induced obesity, exhibited reduced expression of PPARα and carnitine palmitoyltransferase I (CPT-1) in the liver [38]. PPARγ expression is also reduced in PBMC from obese children and adolescents [39]. Indeed, obese mice treated with bezafibrate (BZ), an agonist that preferentially activates PPARα and PPARγ, improves biochemical parameters and reduce the WAT proinflammatory state in obese mice [38]. These findings suggest that obesity in the early stages of life influences the expression of PPARα in leukocytes, and consequently, its reduction can be related to the observed altered lipid profile and reduced insulin sensitivity.

We observed a significant increase in TG and a reduction in HDL levels in obese children and adolescents in comparison with the control group. Both parameters overcome the reference values for pediatric individuals. Noticeably, insulin sensitivity was also affected in the obese group. Thus, we used the HOMA index to evaluate the IR status, which is the most used method for its diagnosis in the pediatric population. The obese subjects displayed a mean value of 4,28, significantly higher than lean subjects, which showed a 1,50 score, predicting IR for the obese group.

As a complementary measure to confirm the prevalence of IR, we calculated the *TG/HDL ratio,* which closely correlated with this parameter in adults at a value ≥3 [40]. This ratio is also considered as a predictor of cardiometabolic risk and reflects an atherogenic lipid profile [41]. In the pediatric population, a high *TG/HDL ratio* is associated with lower insulin sensitivity and cardiovascular risk [42]. We found a significantly increased *TG/HDL ratio* for the obese group (3,02 *p* <  0,0001) in comparison with the lean group (1,60). These results confirm a prevalent condition of IR and cardiovascular risk in obese participants that could be associated with the downregulation of PPARα. Regarding this, PPARα has been reported to improve insulin sensitivity and provide a protective property against the development of IR [37, 43].

Besides, PPARα is recognized by its anti-inflammatory role. In this study, we observed a proinflammatory profile in obese pediatric subjects with an increase in the serum levels of adipsin, MCP-1, IL-8, IL-6, IFN-γ, and TNF-α, and decreased levels of the anti-inflammatory adipokine adiponectin. Hence, PPARα downregulation in leukocytes from obese children suggests an association with their pro-inflammatory cytokine profile. Indeed, PPARα displayed a significant negative correlation with TNF-α levels and the *abdominal skinfold*. TNF-α promotes IR and decreases the uptake of fatty acids by adipocytes, increasing them in circulation while stimulating lipolysis [44, 45].

Furthermore, TNF-α increases the production of inflammatory cytokines such as IL-6 and suppresses the production of adiponectin [45]. TNF-α altogether with IFN-γ and IL-6 leads to an infiltration of immune cells in adipose tissue and dysfunction of inflammatory immune cells [46]. In this work, we found that the concentration of TNF-α was significantly higher in obese patients than in the control group. These findings are consistent with the high HOMA index, IL-8 and IL-6 levels, and the decreased adiponectin concentration in obese children and adolescents.

**Fig. 4:**
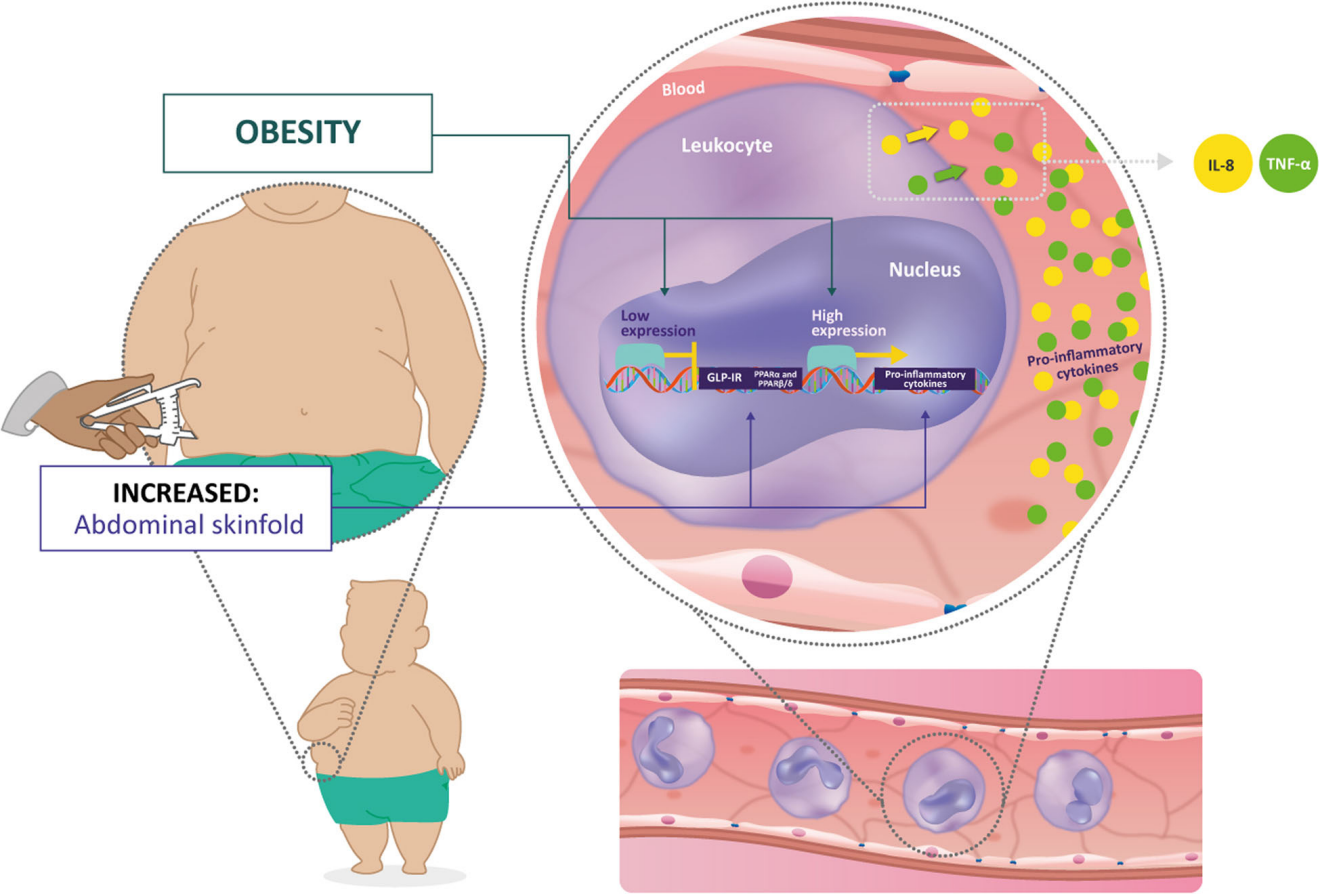
PPARα is negatively correlated with abdominal skinfold and pro-inflammatory markers from leukocytes of an obese pediatric population. Peroxisome proliferator-activated receptor alpha (PPARα) and glucagon-like peptide-1 receptor (GLP-1R) mRNA levels were downregulated in leukocytes from obese children and adolescents. Both receptors were negatively correlated with the abdominal skinfold, indicating its participation in fat distribution. The expression of PPARα and PPARβ/δ was negatively associated with the TNF-α and IL-8 pro-inflammatory markers suggesting a contribution of those isotypes in the modulation of inflammation observed in pediatric obesity. The levels of IL-8 and TNF-α were significantly increased in the serum of obese children and adolescents. Both cytokines are involved in the infiltration of immune cells in adipose tissue and contribute to the inflammatory process

The consequences of obesity on the development of IR and activation of a chronic inflammatory response allowed to establish a relationship between GLP-1R and PPARs. GLP-1 is a hormonal factor that canonically improves insulin secretion on pancreatic β-cells [12, 14]. Otherwise, non-canonical functions of GLP-1R are reported in some lineages of immune cells, such as an immunomodulatory role in the migration and differentiation of T and B lymphocytes [12, 47, 48], and a reduction in monocyte/macrophage migration and inflammatory cytokine production [49, 50]. In this study, we observed a reduced expression of GLP-1R in obese children and adolescents. Although its expression showed no significant association with inflammatory parameters, *GLP-1R* and *PPARα* expression exhibited a negative correlation with *abdominal skinfold* (Fig. 4). These findings suggest a role of both receptors in the adipose tissue distribution that could indirectly affect the role of different immune cells and the secreted cytokines. Besides, GLP-1R and PPARα effects have been associated in pancreatic islets favoring GLP-1R expression and sensitivity to GLP-1 [51], and, in endothelium, leading to downregulation of proinflammatory cytokines [15]. Therefore, both receptors may present a crosstalk in leukocytes from an obese population. This association is highly needed to be explored in the future.

In summary, adipose tissue accumulation in obesity is associated with disturbances in the homeostasis of several tissues, disruption of the mechanisms controlling lipid and glucose metabolism, and dysregulation in serum proinflammatory cytokines and adipokines [46]. It was possible to establish a relationship between anthropometric alterations and body fat distribution observed in obesity with associated comorbidities, such as IR, chronic inflammatory profile, and risk for the development of cardiovascular diseases. In addition, PPARα and GLP-1R represent potential gene targets to be explored in pediatric obesity, based on their roles in abdominal fat distribution and inflammatory profile (Fig. 4).

## Conclusion

We have shown that PPARα and PPARβ/δ expression are negatively associated with the proinflammatory markers TNF-α and IL-8, respectively, suggesting participation in the regulation of inflammation observed in pediatric obesity. Furthermore, PPARα and GLP-1R are downregulated in leukocytes from obese children and adolescents, and both receptors are associated with the abdominal skinfold, suggesting a role in fat distribution that could indirectly affect the function of different immune cells and the secreted cytokines. Thereby, these findings may impact the understanding and implementation of PPARα or GLP-1R agonists in the clinic.

## Materials and methods

### Participants

This is a cross-sectional study with primary data collection performed in children and adolescents from February 2017 to June 201,8 in Bogotá-Colombia. Children and adolescents aged 7 to 17 years old from seven public schools were invited to participate voluntarily but with parental authorization. The study population included participants from low- to middle-income, which represents most of the city population. Finally, 37 participants (female, *n* = 18, and male, *n* = 19), which fulfilled the inclusion criteria, agreed to participate in this study. The inclusion criteria included female and male participants aged 7–17, with a healthy weight (lean control group) and obesity (defined as BMI, ≥ 85th, and ≥ 95th percentile) [52]. Exclusion criteria included: children and adolescents with diabetes mellitus (both type I and II), secondary causes of obesity, psychiatric illness, mental disorders, with weight equal to or greater than 100 kg, as well as those who were receiving medications which promoted weight loss or gain, or which altered in any way the metabolic profile, including glycemia.

The Institutional Review Board of the Antonio Nariño University and the Medical Ethics Committee at DEXA DIAB (CE-CC − 00721) approved the study protocol. Written informed parental consent and child assent from participants were obtained before any research procedures. This study was carried out following the ethics committee code of the American Medical Association endorsed by the National Institutes of Health (NIH).

### Anthropometric assessment

Anthropometric measures were obtained using standard methods [53]. Weight was measured to the nearest 0.1 kg on a calibrated digital scale without shoes and wearing light clothing. Height was measured to the nearest 0.1 cm on a stadiometer. To evaluate the nutritional status of children and adolescents, the BMI Z-score was used, according to the criteria proposed by the World Health Organization [52]. Waist circumference (WC) was measured via flexible measuring tape with an accuracy of 1 mm. The measurement was made with the subject standing, taken at the midpoint between the right lower costal ridge and the iliac crest, without skin compression from the measuring tape, and at the end of a normal expiration. BMI and waist-height were calculated as ratios. Skinfold thicknesses were assessed using a Harpenden Skinfold Calliper, according to a previous protocol [53]. The measurements were performed in triplicate at the non-dominant side on biceps, triceps, subscapular, and suprailiac to the nearest 0.1 mm, and the mean values were calculated [53].

### Measurement of body composition by dual X-ray absorptiometry

Measurements of total and regional body composition were acquired using a Dual-Energy X-ray Absorptiometry (DEXA) scan (GE Lunar Prodigy advance, GE Healthcare) by a trained technician. Before each acquisition, the scanner was calibrated according to the manufacturer’s instructions. The total effective radiation dose during each examination was < 0.05 micro Sievert for a three-minute scan. For analysis, reference data for standard deviation scores were provided from GE Lunar Body composition software (enCORE 2010; version 11.3; GE Healthcare, Madison, WI, USA).

### Biochemical analyses

Blood samples for laboratory analysis were collected by venipuncture from all participants after an overnight fast (10–12 h). Participants underwent a 2-h oral glucose tolerance test (OGTT) (1.75 g/kg, maximum 75 g) according to the American Diabetes Association criteria [54]. Fasting blood samples were obtained for fasting plasma glucose (FPG) insulin levels, total cholesterol, High-density lipoprotein-cholesterol (HDL-c), low-density lipoprotein-cholesterol (LDL-c) VLDL-cholesterol (VLDL-c) and triglycerides (TG). IR was scored using the HOMA-IR index by calculating the product of fasting plasma insulin (U/mL) and fasting plasma glucose (mmol/L) divided by 22.5. TG/HDL ratio was calculated as TG (mg/dL)/HDL (mg/dL) and android-gynoid percent fat ratio was calculated as android fat (%)/gynoid fat (%) (A/G %).

### Leukocyte isolation

An additional tube with an anticoagulant was collected for the isolation of leukocytes by the whole blood lysis method as described previously [55]. The sample was centrifuged at 800 g for 5 min and the leukocyte layer was carefully transferred to a conical tube with Red Blood Cell Lysis Buffer (RBC lysis). The tube was mixed by inversion and incubated for 5 min at room temperature. The samples were centrifuged, and the supernatant was discarded. The procedure was repeated twice. The cell pellet was washed with PBS and preserved in trizol at − 80 °C for RNA extraction.

### Gene expression analysis

Leukocyte total RNA was extracted using Trizol reagent (Life Technologies, Inc) according to the manufacturer’s instructions. RNA was quantified with a Nanodrop ND-1000 spectrophotometer (NanoDrop Technologies), and cDNA was synthesized from 1 μg ARN with the High-Capacity cDNA Reverse Transcription Kit (Thermo Fisher Scientific, Cat. # 4368814).). Real-time PCR was performed in duplicate using CFX96 equipment (BioRad) and SYBR® Green PCR Master Mix, according to the manufacturer’s protocol. *PPAR-α*, *PPAR-β*, *PPAR-γ*, incretins receptor genes *GLP-1R* and *GIPR,* and reference genes *PGK1* and *YWHAZ* were quantitated using the gene-specific primers (Additional file 1). cDNAs were amplified for 40 cycles consisting of 10 s of denaturation at 95 °C, 15 s of annealing temperature for each primer (Additional file 1), and 10 s of extension at 72 °C. Standard curves for all genes were generated using serial dilutions of pooled cDNAs from all samples. Relative mRNA expression was calculated with the method ΔΔCt. Data are shown as normalized ratios between target gene expression and geometric media of the two reference genes [56]. All expression assays were performed following the MIQE guidelines [57].

### Hormone and cytokine levels

Preprandial GIP and GLP1 levels were measured in serum using a specific enzyme-linked immunosorbent assay (Elabscience, Cat. #E-EL-H2061 and E-EL-H6025) according to the procedures provided by the manufacturer. Levels were expressed in ng/mL and pg/mL. The presence of adiponectin, adipsin, RBP4, MCP-1, IL-1β, IP-10, IL-10, IL-8, IL-6, IFN-γ, resistin, and TNF-α in serum samples was analyzed using a multiplex immunoassay based on fluorescence-encoded beads according to the manufacturer’s instructions (No. 40196, BioLegend). The acquisition was performed in an Accuri C6 flow cytometer. Off-line analysis was performed with LEGENDplex™ data analysis software (version 8.0), and the data were expressed as the mean reporter fluorescence intensity PE (MFI) as a function of concentration (pg/mL). Each assay was performed with two technical replicates.

### Statistical analysis

Statistical analysis was performed using GraphPad Prism 6. Descriptive statistics were expressed as mean ± standard deviation (SD) whenever applicable, and variables were tested for normality by Kolmogorov-Smirnov test. Non-parametric tests were used whenever data were not normally distributed or Student *t*-test when data were normally distributed. We used Wilcoxon signed-rank tests to assess the significance of inter-group differences. The correlation of target genes and evaluated parameters were determined using the Spearman rank test. For all analyses, statistical significance was considered as *p* <  0.05.

## Supplementary Information

**Additional file 1.** Sequences of primers used for real time qPCR.

**Additional file 2.** Correlation of DEXA fat measures and anthropometric data with metabolic risk factors.

**Additional file 3.** Anthropometric, body composition and biochemical parameters categorized by gender.

**Additional file 4.** Anthropometric, body composition and biochemical parameters categorized by age.

## Data Availability

The datasets used and analyzed during the current study are available from the corresponding author on reasonable request.
