# Supplementary material for: PPARα and PPARβ/δ are negatively correlated with proinflammatory markers in leukocytes of an obese pediatric population
Source: J Inflamm (Lond). 2020 Oct 31;17:35. doi: 10.1186/s12950-020-00264-2 (PMC7602348; doi:10.1186/s12950-020-00264-2)
Supplement: Supplementary file 1 — Additional file 1. Sequences of primers used for real time qPCR. [file 12950_2020_264_MOESM1_ESM.pdf]

Additional file 1. **Sequences of primers used for real time qPCR**

**Access number**	**Gene**	**Primer sequence**	**Ta °C**	**Amplicon size (bp)**	**Efficiency (E) (%)**	**Correlation coefficient (R^2)**
NM_001001928.3	PPAR-α	F AGCTGTCACCACAGTAGCTTG<br>R CAGAGTGGGCTTTCCGTGTC	58	82	95,72	0.996
NM_001171818.2	PPAR-β	F ACCAACGAGGGTCTGGAATG<br>R AGCCTTGAAGCAGTCCTGTA	58	108	96,72	0.956
NM_001354666.3	PPAR-γ	F GGCATCCCCCTAAACTTCG<br>R TGGCTTCTTTCAAATCTGGTG	58	106	94,13	0.967
NM_002062.5	GLP-1R	F AGAAATGGCGAGAATACCGAC<br>R TTCATCGAAGGTCCGGTTG	60	95	102,955	0.950
NM_000164.4	GIPR	F TGCCCTTCTGGAGATGACAAC<br>R TTCTCGCTTCCCTTCATAACCA	58	100	111,365	0.992
NM_000291.4	PGK1	F CGGGTCGTTATGAGAGTCG<br>R AATTTGATGCTTGGGACAGC	60	95	90,320	0,977
NM_001135699.1	YWHAZ	F CATCACTCAGCCACACTCAGG	58	100	96,530	0,982

		R ATGACTGGATGTTCTGCTGGCTC				

*F: forward primer; R: reverse primer; Ta: Annealing Temperature.
